# What Factors Explain Low Adoption of Digital Technologies for Health Financing in an Insurance Setting? Novel Evidence From a Quantitative Panel Study on IMIS in Tanzania

**DOI:** 10.34172/ijhpm.2023.6896

**Published:** 2023-02-13

**Authors:** Leon Schuetze, Siddharth Srivastava, Naasegnibe Kuunibe, Elizeus Josephat Rwezaula, Abdallah Missenye, Manfred Stoermer, Manuela De Allegri

**Affiliations:** ^1^Heidelberg Institute of Global Health, Medical Faculty and University Hospital, University of Heidelberg, Heidelberg, Germany.; ^2^Swiss Tropical and Public Health Institute (Swiss TPH), Basel, Switzerland.; ^3^University of Basel, Basel, Switzerland.; ^4^Faculty of Integrated Development Studies, University for Development Studies, Wa, Ghana.; ^5^Health Promotion and System Strengthening Project (HPSS), Dodoma, Tanzania.; ^6^Kongwa District Council, Dodoma, Tanzania.

**Keywords:** Health Financing, Health Insurance, Strategic Purchasing, Tanzania, Digital Health Intervention, Adoption

## Abstract

**Background:** Digital information management systems for health financing are implemented on the assumption thatdigitalization, among other things, enables strategic purchasing. However, little is known about the extent to which thesesystems are adopted as planned to achieve desired results. This study assesses the levels of, and the factors associated withthe adoption of the Insurance Management Information System (IMIS) by healthcare providers in Tanzania.

**Methods:** Combining multiple data sources, we estimated IMIS adoption levels for 365 first-line health facilities in2017 by comparing IMIS claim data (verified claims) with the number of expected claims. We defined adoption as abinary outcome capturing underreporting (verified<expected) vs. not-underreporting, using four different approaches.We used descriptive statistics and analysis of variance (ANOVA) to examine adoption levels across facilities, districts,regions, and months. We used logistic regression to identify facility-specific factors (ie, explanatory variables) associatedwith different adoption levels.

**Results:** We found a median (interquartile range [IQR]) difference of 77.8% (32.7-100) between expected and verifiedclaims, showing a consistent pattern of underreporting across districts, regions, and months. Levels of underreportingvaried across regions (ANOVA: F=7.24, *P*<.001) and districts (ANOVA: F=4.65, *P*<.001). Logistic regression resultsshowed that higher service volume, share of people insured, and greater distance to district headquarter were associatedwith a higher probability of underreporting.

**Conclusion:** Our study shows that the adoption of IMIS in Tanzania may be sub-optimal and far from policy-makers’expectations, limiting its capacity to provide the necessary information to enhance strategic purchasing in the healthsector. Countries and agencies adopting digital interventions such as openIMIS to foster health financing reform areadvised to closely track their implementation efforts to make sure the data they rely on is accurate. Further, our studysuggests organizational and infrastructural barriers beyond the software itself hamper effective adoption.

## Background

**Key Messages**

** Implications for policy makers**

- Digital information systems like the Insurance Management Information System (IMIS) are seen as instrumental to the implementation of strategic purchasing, which in turn is considered a key element towards achieving universal health coverage.
- In the studied setting, IMIS adoption by healthcare providers was very low across regions, districts, and facilities, rendering it inappropriate to base purchasing decisions on the data it provides.
- While district management, workload, and remoteness of facilities seem to be influencing IMIS adoption, it is likely that other contextual factors beyond those investigated here play a major role as well. Further research is needed to investigate root causes of low adoption to achieve effective strategic purchasing.
- Policy-makers and agencies adopting digital information systems for strategic purchasing should closely track and evaluate their implementation efforts to ensure the data they rely on are accurate.

** Implications for the public**

Advancing sustainable health systems to foster universal health coverage is in the best interest of the public. Strategic purchasing can only help achieve this if the tools used to inform purchasing decisions are well designed and implemented. By investigating the implementation of one of these tools, the Insurance Management Information System (IMIS/openIMIS), we raise the concern that this might not automatically be the case. Adoption of the software by first-line healthcare providers was sub-optimal, risking the success of the whole implementation. A scientific public sensitized for this issue can play an important role in future implementation efforts, by thoroughly tracking and evaluation their success, and aiding implementers and policy-makers with their findings.

Moving towards universal health coverage often requires that substantial reforms are implemented across all three health financing functions. Namely, these are resource generation (where and how funds are collected), resource pooling (how funds from multiple sources are combined to share the financial risk of paying for healthcare), and purchasing (how purchasing agents purchase health services from healthcare providers).^1^ The Lancet Commission on High Quality Health Systems in the Sustainable Development Goal Era acknowledged that out of these health financing functions, purchasing has the greatest influence on the quality of service delivery.^2^ While passive purchasing is characterised by fixed budget allocations and payments independent of performance, strategic purchasing refers to how a purchaser, eg, a ministry of health or an insurance scheme, makes strategic and well-informed decisions on: (*a*) which health services to purchase; (*b*) which providers to purchase from; and (*c*) how to purchase services.^3,4^ The benefits and effects that strategic purchasing can yield have been described in-depth.^5,6^ To list just the key ones, strategic purchasing is expected to foster accountability, increase efficiency, and ultimately improve quality of care. The World Health Organization (WHO) identifies moving from passive to strategic purchasing as a pivotal element guiding health financing reforms towards universal health coverage and as a means of establishing more efficient health systems.^7,8^

 Adopting strategic purchasing solutions in practice, however, is a complex endeavour. The literature indicates that in addition to political will, financial ability, and technical know-how, access to robust and comprehensive information systems is crucial.^2,5,7^ Strategic purchasing decisions can only be made based on accurate knowledge of both a population’s demographics and its health needs and providers’ capabilities.^7^ It follows that strong information systems supporting knowledge management are a requirement for the implementation of strategic purchasing mechanisms.^5^

 Information systems, as routinely used in high-income settings, are considered a viable means of enhancing knowledge management in low resource settings as well.^9^ Yet, only few digital interventions have been developed specifically targeting strategic purchasing.^1^ This is surprising considering that digital interventions for health financing, such as openRBF^10^ and openIMIS,^11^ yield vast potential to support a shift towards strategic purchasing, by providing a comprehensive base of health and facility data and handling complex payments tied to a range and combination of performance parameters (eg, capitation systems, payment weights, etc).

 The Insurance Management Information System (IMIS) is one of a handful of software specifically developed to support management of health financing schemes, specifically health insurance, in all of its business procedures (ie, enrolment, renewal, claims, feedback, reporting).^11,12^ IMIS was initially developed by the Swiss Tropical and Public Health Institute to support the ‘improved Community Health Funds’ (iCHF) in Tanzania. It was then released as an open-source software (openIMIS) and implemented in multiple countries.^11,13,14^ One core feature of openIMIS is the bringing together of provider and beneficiary data, allowing comprehensive knowledge management, and hence facilitating the implementation of strategic purchasing mechanisms. The key component enabling strategic purchasing is the claim management function. For each patient contact, facility staff should enter a claim in the system to be reimbursed by the insurer, binding payments to specific outputs as opposed to predefined inputs. Hence, claim data can enable further strategic purchasing decisions.

 While some evidence is emerging to document experiences with the implementation of routine health information systems or other digital health interventions,^15-21^ to our knowledge, very limited data on the implementation of digital interventions specific to strategic purchasing and more generally to health financing is available in the scientific literature. In a recent evidence review on digital financial services for health by the United States Agency for International Development, the vast majority of references stemmed from project reports rather than the peer-reviewed literature and covered almost exclusively mobile money services or similar tools to improve resource generation and pooling.^22^ This means that policy-makers are investing in the implementation of digital interventions for health financing with limited understanding of their effective reach, the barriers and facilitators to their adoption nor any insight into stakeholders’ views.^23^

 This study addresses this knowledge gap. Our objective was to assess levels and identify determinants of adoption^24^ of the IMIS claim management function by public first-line facilities in Tanzania. Our ambition is to contribute initial evidence to inform further implementation of digital interventions for health financing, especially for strategic purchasing, in resource-limited settings.

## Methods

###  Insurance Management Information System in Tanzania

 IMIS was first implemented as a management software for the iCHF in the region of Dodoma in 2012 and expanded to Morogoro and Shinyanga in 2014/2015. As every public healthcare facility in those regions handled iCHF clients, all facilities were required to work with IMIS.

 IMIS claims could be entered either via a laptop or a mobile phone and could be uploaded whenever an internet connection was available. For every IMIS claim, the paper claim sheet that was used before the introduction of IMIS had to be filled as well, resulting in a double reporting mechanism. Claim reimbursements were only calculated based on IMIS claims, however, not on paper claims. In some facilities, especially in early months of the implementation, claims were not entered by facility staff, but by district staff after submission of paper claim sheets. Facility staff were supposed to receive an initial training by a project team or the regional iCHF management. Further support was organized through district coordinators and the district IT. During monthly supportive supervision visits by the council health management team, paper claims could be collected and cross-checked with IMIS claims. There is no data available on the implementation of these training and supervision procedures.

###  Study Design, Study Population, and Sampling

 This observational study made use of IMIS facility-specific monthly claim counts to construct a retrospective longitudinal analysis of claims data for each month in 2017. More specifically, we measured the adoption of the IMIS claim function as the discrepancy between the number of reimbursement claims actually processed through IMIS (verified claims) and the expected number of claims in a panel of 365 first-line public facilities^25^ located across the three iCHF implementation regions (Dodoma, Morogoro, Shinyanga) and we explored factors associated with this discrepancy. Every public first-line facility with available data for 2017 in both IMIS and the district medical records was included. Out of an original census of 449 facilities, 84 had to be excluded due to either unspecific data errors (n = 13) or missing values on key variables of interest in >4/12 months (n = 71), leaving a sample of 365.

###  Variables and Their Measurement

 To define outcome and explanatory variables, we combined data across multiple data sources. Table 1 illustrates the data extracted for analysis and the corresponding source. Table 2 provides a list of outcome and explanatory variables, including their measurement and the direction of the expected association with the outcome variables.

**Table 1:** Data and Their Sources

**Data Extracted**	**Data Source**	**Explanation**
Number of people insured by village Share of people insured by village Claims per month by facility	AR-IMIS	IMIS component allowing extraction of any operational data from the IMIS data warehouse
Utilization rate by region; iCHF insured vs all respondents	DHS Tanzania 2014/2015	DHS Tanzania conducted in 2014/2015
Facility staffing levels Facility setting (rural/urban) Outpatient service volume per month by facility Facility catchment area	DMR	District paper record data received from district medical officers by HPSS project and IT officers
Percentage of health service utilization allocated to public dispensaries and health centres; iCHF insured vs all respondents	HPSS household survey 2018	Household survey conducted in 2018 to measure project achievements established in the first project phase. 1469 households in 7 districts in Dodoma answered a questionnaire about (among other things) iCHF and health service utilization
Facility ownership (public vs. private/missionary) GPS location of health facilities	Tanzania HFR	Publicly available government web portal containing approved information about all health facilities in Tanzania
GPS location of district administration offices (district headquarter/iCHF coordinator)	HPSS project^a^	Provided by local project staff

Abbreviations: IMIS, Insurance Management Information System; AR-IMIS, IMIS analytic and reporting component; DHS, Demographic and Health Survey; DMR, district medical records; HFR, health facility registry; iCHF, improved Community Health Funds; HPSS, Health Promotion and System Strengthening; IT, information technology; GPS, Global Positioning System. ^a^HPSS project in which iCHF is embedded, mandated by the Swiss Agency for Development and Cooperation (SDC), and implemented by Swiss Tropical and Public Health Institute (TPH).

**Table 2:** Variables and Their Measurements

**Variable**	**Definition**	**Source(S)**	**Measurement**	
**Outcome variable**				
Underreporting	Observation classified as underreporting for respective model (1-4)	See formula (1) and formula (2) - AR-IMIS - DHS - DMR - HPSS household survey	1 if underreporting 0 if otherwise	
**Explanatory variables**				*Expected association*
No of staff	Staffing level at facility	DMR	Count	Reduce underreporting
Service volume	Outpatient contacts per month, all patients	DMR	Count	Increase underreporting
Distance to headquarter	Linear distance between facility and district iCHF coordinator office	HFR HPSS project	Continuous	Increase underreporting
Share insured	Share of population insured via iCHF in the facility catchment area	AR-IMIS	Continuous	Both ways possible

Abbreviations: IMIS, Insurance Management Information System; AR-IMIS, IMIS analytic and reporting component; DHS, Demographic and Health Survey; DMR, district medical records; HFR, health facility registry; iCHF, improved Community Health Funds; HPSS, Health Promotion and System Strengthening.

###  Outcome variable

####  Estimation of Expected Claims

 A unique feature of our work is that prior to our analysis, we had to compute a tangible outcome variable to allow us to capture the facility-specific monthly discrepancy between expected and actual claims. To do this we first had to compute the number of expected claims per observation. Using data on health facility specific service volume and the number of people insured via the iCHF in the facility catchment area, we assumed that the number of expected visits for each facility could be approximated by counting only the share of visits represented by the number of iCHF insured in the catchment area. However, it is known that health service utilization of people with insurance can be higher. Therefore, we included in our calculation a term that corrects for differences in utilization between iCHF insured and not insured. Combining in a single matrix information from multiple sources, this resulted in the following formula:


(1) $$expected_{fm}=visits_{fm}\times insured_{fm}\times \frac{utilization_{CHF}}{utilization_{all}}$$

where *expected*_fm_ is the number of expected claims for facility *f* in month *m*, *visits*_fm_ is the service volume of the facility, ie, the number of all outpatient contacts, not only iCHF patients, and *insured*_fm_ is the percentage of people insured in the facility catchment area. *utilization*_CHF_ and *utilization*_all_ are the utilization rates for iCHF insured and the overall population in the facility’s region respectively, and account for the expected higher utilization rate of insured people compared with the general population. Utilization rates were computed for each region using publicly available data^26,27^; *insured*_fm_ was computed by combining data sources on village population, the number of insured per village and the facility catchment area (computation details in Supplementary file 1).

####  Calculation of Outcome Variable

 We calculated the relative difference between expected and verified claims for each observation and expressed it as a percentage, using the formula^28^:


(2) $$D_{fm}=\frac{expected_{fm}-verified_{fm}}{expected_{fm}}\times 100$$

where *expected*_fm_ is the number of claims expected for facility *f *in month *m *and *verified*_fm_ is the number of claims observed in IMIS. Positive values represent fewer verified than expected claims (underreporting), while negative values represent the opposite (overreporting).

 Since preliminary analysis revealed overreporting to be a lot less common than underreporting, we focused our multivariate analysis on underreporting. Because the data set did not meet assumptions for linear regression, we constructed our regression outcome as 1 = underreporting and 0 = not underreporting. We constructed 4 models that only differed in the setting of the outcome to verify robustness of results to different conceptional definitions of what represents underreporting.Following Kuunibe et al,^28^ we set an underreporting threshold in several ways: first, relying on formula (2), we classified as underreporting all observations where the discrepancy between expected and verified equalled or exceeded 10% (model 1), 25% (model 2) and 50% (model 3). Second (model 4), we used the median absolute difference (MAD) as described by Leys et al^29^ and defined our underreporting threshold accordingly. To calculate the MAD, the median of the data set is subtracted from all values, and the resulting median of the new values is then multiplied by a coefficient depending on the distribution of the data set. This represents a more robust method to detect outliers than using deviation from the mean, as it is not itself influenced by the presence of outliers. All observations with >2.5*MAD deviation were classified as underreporting (=1), whereas all observations within 2.5*MAD and -2.5*MAD were classified as not underreporting (=0).

###  Explanatory Variables

 As described in Table 2, explanatory variables included staffing level, service volume, share of people insured via iCHF in the catchment area, and distance from the facility to the iCHF coordinator.^30^ Explanatory variables were chosen based on the below-mentioned conceptual ideas derived from the literature and data availability.

 We hypothesize that facilities with less staff struggle more to keep up with documentation, as each staff member has to handle more work individually, especially considering that understaffing is a severe issue in the Tanzanian health system.^31,32^ Service volume (eg, outpatient contacts per month) was used as a determinator of workload. Considering the shortage of staff in most facilities, we expect a higher workload to increase underreporting. As a measure of the overall success of the iCHF scheme in the catchment area of a facility, we used the share of people insured via iCHF in the catchment area. A higher percentage of insured could imply better management of claims, but could also lead to an overwhelming amount of iCHF patients resulting in underreporting.

 Close supervision and on-job training are both factors that can influence data quality in a health information system^33^ and facility remoteness can lead to a reduced number of supervision visits.^34,35^ Preliminary qualitative information during this study suggest that the same is the case with IMIS in Tanzania, prompting the expectation that underreporting could be a more severe problem for remote facilities. Since the number of supervision visits for each facility was not available or feasible to retrieve, the distance of the health facility to their district iCHF coordinator (eg, supervisor) was used as a proxy measurement for supervision.

###  Missing Values

 Missing values for explanatory variables or underlying data ranged from 0% (distance to headquarter) to 8.8% (outpatient service volume). Other variables/data with missing values included the facility catchment population (3%), the number of people insured in the catchment area (2.2%) and staffing levels (1.6%). We relied on single imputation, imputing the district median value for variables with a skewed distribution and the district mean for variables with a normal distribution. During the estimation of expected claims, data had to be imputed on several levels. For imputation of outpatient service volume, seasonal variation of utilization was considered as well, using the following formula:


(3) $$p=\frac{{µ}_{f}}{{µ}_{d}/{µ}_{dm}}$$

where p is the missing value, µ_f_ is the mean service volume of the facility in all available observations, µ_d_ is the mean service volume in the district, and µ_dm_ is the mean service volume in the district in the respective month of the missing value.

###  Analytical Approach

 Our analysis proceeded in stages. First, we used descriptive statistics to describe the discrepancy between expected and verified claims (our outcome measure of misreporting) in terms of both absolute numbers and percentages across facilities, districts, and months. We also used descriptive statistics to explore the distribution of outcome (underreporting) and explanatory variables. Second, we relied on a one-way analysis of variance (ANOVA) to test if the reporting quality differed significantly across districts and regions. Third, to explore factors associated with underreporting, we used logistic regression. Given the multilevel structure of the data, we relied on a mixed effects model specifying as fixed effects the abovementioned explanatory variables and as random intercepts both district and facility effects.


(4) $$\begin{array}{l} y_{fm}^{*}=X_{fm}ß+Z_{fm}u_{m}+\in_{fm};\;f=1,...,n;\;m=1,...,T_{i}\\ y_{fm}=1\;if\;y_{fm}^{*}>0,\;and\;0\;otherwise, \end{array}$$

where $\left(y_{fm}^{*}>10/25/50\right)$ for models 1-3 for facility *f *in month *m* and $\left(y_{fm}^{*}>2.5MAD\right)$ for facility *f* in month *m* for model 4. y_fm_ is underreporting for facility *f* in month *m*; X_fm_ is a vector of explanatory variables; ß is a vector of coefficients; and $\in_{fm}$ is the random error term.

 For model 4, all observations below -2.5*MAD, ie, overreporting outliers, were dropped, since detected outliers cannot conceptually be pooled with non-outliers. This results in the model 4 outcome being underreporting vs. right-reporting while for models 1-3 it is underreporting vs. not underreporting.

 We performed several sensitivity analyses: (*a*) applying a pooled utilization rate for all regions instead of regional utilization rates; (*b*) using a different method of computing expected claims, relying solely on the number of insured and utilization rates, not considering the number of visits; (*c*) without imputation of missing values. Analysis was performed using Stata 15.

## Results

###  Descriptive Statistics

 The 365 facilities in the three regions were spread across 19 districts, ranging from four to 38 per district with a mean of 19. With 188 (51.5%) facilities, more than half of the facilities were located in Dodoma region, followed by Shinyanga (n = 101; 27.7%) and Morogoro (n = 76; 20.8%).

 All explanatory variables showed highly heterogeneous values. The median (IQR) number of staff was 4 (3-5), ranging from 1 to 25. The mean (standard deviation) service volume was 449 (427.9) outpatient contacts per month, ranging from 3 to 7194. The median (IQR) distance to the CHF coordinator was 33.2 km (16.9-52.5 km), ranging from 0.5 km to 156.5 km and the median (IQR) number of people insured in the catchment area was 12.4% (6.4%-20.1%) with a range from 0-98.8%. A table with summary statistics for the explanatory variables is reported in Supplementary file 2 (Table S2).

 The number of verified and expected claims differed between regions, with the median (IQR) number of verified claims ranging from 5 (0-31) in Dodoma to 27 (3-51) in Shinyanga, and the median (IQR) number of expected claims ranging from 34 (13.9-73) in Dodoma to 89.7 (44.2-189.4) in Shinyanga. We observed a median (IQR) difference of 77.8% (32.7%-100%) between expected and verified claims, ranging from 83.4% (26.6%-100%) in Dodoma to 75.9% (48.1%-96.5%) in Shinyanga and 75.3% (18%-100%) in Morogoro, respectively. Supplementary file 2 (Table S3) provides numbers for all regions and districts. The percentage difference was lower in July and August, but no apparent improvement or deterioration in reporting was observed over the course of the year (Figure).

**Figure:**
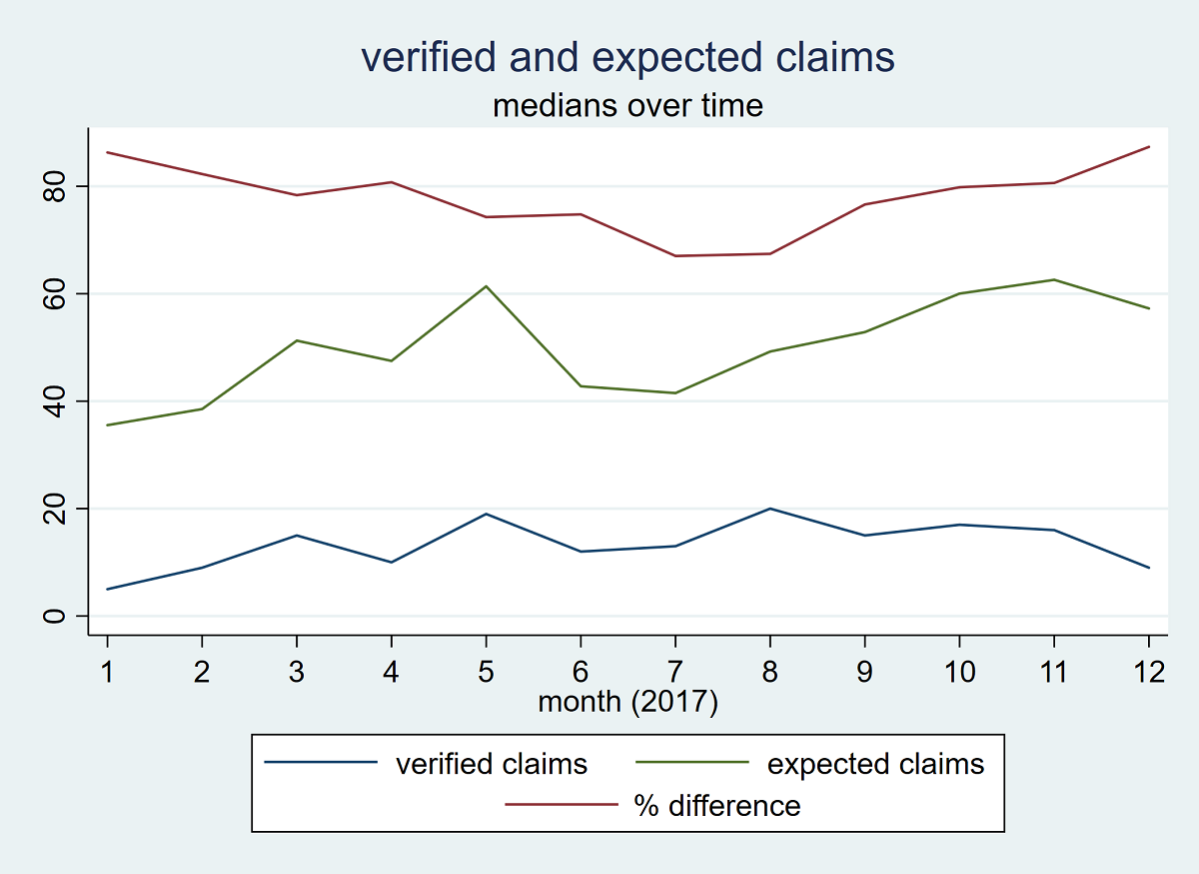
Verified and Expected Claims Over Time.

 Descriptive analysis of the outcome variable (Table 3) showed that for the 10%, 25%, and 50% thresholds (models 1-3), the majority of observations were classified as underreporting. Morogoro consistently presented the lowest number of observations classified as underreporting, with 78%, 73%, and 65%, respectively. Dodoma followed with 79%, 75%, and 65%, while Shinyanga presented the highest numbers with 90%, 85%, and 74%, respectively. Heterogeneity between districts was even higher, ranging from 61% to 100% applying the 25% threshold. A different pattern was observed when defining the outcome in relation to 2.5*MAD (model 4). The number of observations classified as underreporting was not only lower with a district range from 2% to 89%, but the regional order also changed. Dodoma had the lowest number (15%), followed by Morogoro (34%) and Shinyanga (47%).

**Table 3:** Descriptive of Outcome Variables (After Imputation)

**Outcome**	**Percentage Misreporting**			**Observations**
	**Under**	**Over**	**No**	**N**
(1) 10% difference	82	14	4	4.279
(2) 25% difference	78	12	10	4.279
(3) 50% difference	68	9	23	4.279
(4) 2.5*MAD	28	1	71	4.380

Abbreviation: MAD, median absolute difference.

 The one-way ANOVA showed that there was a statistically significant difference between districts (F(18, 4260) = 4.65, *P* <.001) and regions (F(2, 4276) = 7.24, *P* < .001). A Tukey post-hoc test revealed that underreporting was significantly higher in Shinyanga than in Dodoma (76.2 ± 17.91 packages, *P* =.001) while differences between Shinyanga and Morogoro or Dodoma and Morogoro were not statistically significant.

###  Regression Results

 Results of the logistic regression are presented in Table 4. A total of 4279 (models 1-3) to 4321 (models 4) observations were included in the model. The direction of associations was consistent across all models. The size of the coefficients was consistent in models 1-3, but differed in model 4.

**Table 4:** Logistic Regression Results

	**(1) 10% Threshold​**	**(2) 25% Threshold​**	**(3) 50% Threshold​**	(4) 2.5*MAD​
**Explanatory variables​**				
No. of staff (95% CI)	0.050 (-0.048–0.154)	0.052 (-0.043–0.147)	0.077 (-0.009–0.162)	0.082 (-0.043–0.207)
*P* value	.303	.283	.078	.199
Service volume (95% CI)	0.002 (0.001–0.002)	0.002 (0.001–0.002)	0.001 (0.001–0.002)	0.008 (0.007–0.009)
*P* value	<.001	<.001	<.001	<.001
Distance to headquarter (95% CI)	0.014 (0.005–0.023)	0.012 (0.003–0.020)	0.014 (0.006–0.022)	0.000 (-0.013–0.013)
*P* value	.002	<.001	<.001	.980
Share insured (95% CI)	0.084 (0.064–0.104)	0.073 (0.056–0.090)	0.053 (0.039–0.067)	0.268 (0.237–0.298)
*P* value	<.001	<.001	<.001	<.001
Wald Chi^2^	109.1	110.7	101.2	373.8
*P* value	<.001	<.001	<.001	<.001
**Random intercepts**				
District (95% CI)	0.415 (0.092–1.860)	0.354 (0.070–1.800)	0.167 (0.023–1.222)	2.713 (1.162–6.332)
Facility (95% CI)	2.586 (1.957–3.416)	2.692 (2.071–3.500)	2.849 (2.243–3.620)	3.638 (2.564–5.162)
N​	4279	4279	4279	4321

Abbreviations: MAD, median absolute difference; CI, confidence interval.

 A higher service volume and share of people insured in the catchment area were associated with a higher probability of underreporting (*P* < .001), the same for greater distance to district HQ in model 1-3 (*P* < .01). The number of staff was not associated with reporting behaviour.

 Results of the sensitivity analysis (Supplementary file 3) confirmed that findings were largely robust to variations in the parameter estimates.

## Discussion

 Our study makes a substantial contribution to the scientific literature, by being, to the best of our knowledge, the first to examine the adoption and the determinants of adoption of a digital interventions to enhance the strategic purchasing function of a health financing innovation, specifically of a health insurance scheme, in sub-Saharan Africa. Prior work in similar settings had only focused on the implementation of routine health information systems or other tools unrelated to health financing.^15-21^ Although we recognize important methodological limitations, we do trust that our study offers important insights into the extent to which the adoption of digital interventions for health financing may fall short of the expectations entrusted upon it and why. As such, our study provides valuable information for policy-makers committed to advancing the adoption digital interventions for health financing in resource-limited settings. This information is of particular interest considering the push towards large-scale implementation that openIMIS is subject to among policy-makers.

 The first striking finding is the low level of adoption observed in our study. Across facilities, districts, and regions, with a median of 77.8%, the discrepancy between expected and verified claims was extremely high. This indicates that the low adoption of IMIS is a widespread problem in Tanzania, cutting across facilities, districts, and regions, and suggests the existence of a systemic problem. This low adoption is surprising considering that payments to the facility depend on their claims. Nonetheless, some considerable heterogeneity exists. In some instances, districts bordering each other displayed drastically different reporting quality, and even within many districts, the median percentage difference between facilities differed by over 50%. While further qualitative research is needed to understand why facilities would forgo claiming for services provided and to investigate sources of heterogeneity across districts/regions, our analysis already suggests some explanatory factors.

 First, differences in IMIS adoption across districts could be explained by the fact that the management of the iCHF is organized at the district level. Not only is the quality of management known as a key predictor for the successful implementation of ICTs in health,^18,33^ but district management has also been observed to be an important factor in shaping overall health system performance in many settings.^36,37^ Second, confirming the hypothesis that management styles do play an important role in shaping the adoption of digital innovations, our findings revealed that an increased distance to the iCHF coordinator increased the probability of underreporting. This finding is well aligned with what has been reported in other settings^20,35^ and suggests that supportive supervision represents a key factor for the successful implementation of innovations in health. It is very likely that remote facilities received fewer visits from iCHF coordinators and as such developed a limited capacity to comply with IMIS requirements. While further qualitative research exploring the matter is needed, this pattern is likely to be exacerbated by the high turnover that rural facilities experience in sub-Saharan settings.^38-40^ It is possible that providers trained at the onset of an intervention move out of a given facility, leaving new providers to manage a system they have never been trained for. Third, the fact that facilities with a higher service volume displayed lower adoption levels is not surprising and well aligned with prior research describing workload as a key barrier to the adoption of digital interventions or other innovations in health.^23,33,41^ This suggests that sufficient human resource capacity needs to be available to enable the implementation of innovations such as digital interventions in the health sector. We advance the hypothesis, to be confirmed by further research, that the parallel introduction of iCHF and a performance-based financing program^42^ relying on its own digital reporting system might have been especially challenging for high-volume facilities in one region. The challenge imposed by the additional workload that comes with implementing health system innovations has been reported before, primarily in the qualitative literature on performance-based financing,^43-45^ but it is likely to apply to iCHF management as well. Should further research confirm the veracity of our hypothesis, policy-makers should consider integrating reporting systems across programs to reduce administrative workload on providers. Surprisingly, however, we did not note an association between staffing levels and underreporting. In this regard, one needs to consider that throughout Tanzania, first-line facilities are understaffed compared with government staffing level plans.^25,31,32^ This means that differences in staffing levels in our sample are probably negligible and unlikely to affect the outcome of interest.

###  Methodological Considerations

 Beyond its strengths, we need to acknowledge a few methodological limitations of this study. First, in the absence of policy-given or evidence-based thresholds, we applied arbitrary thresholds to define our measure of underreporting. Nonetheless, we recognize that results are largely consistent across models, reinforcing the robustness of our key findings and suggesting that policy-makers select the most relevant threshold to inform their decisions. Second, we acknowledge that data availability constrained the range of explanatory variables included in our analysis. Potentially interesting explanatory variables, such as claim entry method (online vs offline) or opening year had to be excluded owing to data quality concerns. While omitted variable bias could have affected the size of the model coefficients for the included variables,^46^ it does not affect the key finding detecting widespread low adoption. Third, with regard to the generalizability of the findings, we have to acknowledge that the three implementation regions were chosen by the implementer mainly for practical reasons and not primarily to be representative of the country at large. Finally, in the absence of any data measuring actual service provision to iCHF insured patients, we had no choice, but to rely on an estimated measure of expected service delivery against which to assess IMIS reporting. While we are aware that no estimation can ever capture reality fully, we trust in the validity of the measure, since we took every possible measure to guarantee that our estimation was as reasonably close as possible to reality. The trustworthiness of our analysis is further confirmed by the findings of the sensitivity analysis.

## Conclusion

 Implementing strategic purchasing approaches relies on the availability and widespread adoption of reliable information systems for data collection and management. Our study suggests that in the case of the iCHF, IMIS adoption may be sub-optimal and far from policy-makers’ expectations. Whether due to weak district management, high service volumes/high staff workload, higher distance to the district coordinator (leading to less supervision) or other, unobserved reasons, low adoption results in the generation of a poor database that limits capacity to provide the necessary information to further enhance strategic purchasing in the health sector.

 Our findings indicate that countries and agencies adopting digital interventions for health financing such as openIMIS to enable strategic purchasing and foster health financing reforms need to consider specific contextual elements potentially hampering the effectiveness of such systems. Agencies adopting digital interventions are advised to track closely and scientifically evaluate their implementation to make sure the data they rely on are accurate. In line with prior evidence,^21,23,47-51^ our study suggests organizational and infrastructural barriers beyond the software itself hamper effective utilization. Further qualitative research^52^ is necessary to examine in greater depth the reasons behind the low adoption of digital interventions for health financing.

## Acknowledgements

 We acknowledge the support by the Else Kröner-Fresenius-Stiftung within the Heidelberg Graduate School of Global Health. We are grateful to Salvador Shabbir for his support in language editing.

## Ethical issues

 Since the study relied exclusively on fully anonymized secondary data, we received a waiver for ethical approval from the Ethics Committee of the Medical Faculty of the University of Heidelberg. We obtained ethical approval from the National Institute for Medical Research (NIMR) in Tanzania (NIMR/HQ/R.8a/Vol.IX/3031).

## Competing interests

 MDA and NK declare no competing interests. SS and MS are part of the Swiss TPH project team that is implementing HPSS project under which openIMIS was implemented. ER is a project advisor for HPSS project. LS has a doctoral student agreement with and received travel support by Swiss TPH. AM has performed paid activities with HPSS project in the past.

## Authors’ contributions

 Conception and design: LS, MDA, and SS. Acquisition of data: LS, SS, EJR, and AM. Analysis and interpretation of data: LS, SS, and MDA. Drafting of the manuscript: LS and MDA. Critical revision of the manuscript for important intellectual content: SS, EJR, AM, NK, and MS. Statistical analysis: LS and NK.

 Administrative, technical, or material support: SS, EJR, and MS. Supervision: MDA and SS.

## Funding

 For the publication costs we acknowledge financial support by Deutsche Forschungsgemeinschaft within the funding programme “Open Access Publikationskosten” as well as by Heidelberg University.

## Supplementary files


Supplementary file 1. Calculation of Values to Estimate Outcome.

Supplementary file 2. Descriptive Statistics.

Supplementary file 3. Sensitivity Analysis.

## References

[1] Meessen B (2018). The role of digital strategies in financing health care for universal health coverage in low- and middle-income countries. Glob Health Sci Pract.

[2] Kruk ME, Gage AD, Arsenault C (2018). High-quality health systems in the Sustainable Development Goals era: time for a revolution. Lancet Glob Health.

[3] Honda A. What is Strategic Purchasing for Health? RESYST; 2014. https://assets.publishing.service.gov.uk/media/57a089c8e5274a27b2000271/Purchasing-brief_web.pdf.

[4] Figueras J, Robinson R, Jakubowski E. Purchasing to Improve Health Systems Performance. McGraw-Hill Education (UK); 2005.

[5] Busse R, Figueras J, Robinson R, Jakubowski E (2007). Strategic purchasing to improve health system performance: key issues and international trends. Healthc Pap.

[6] Tangcharoensathien V, Limwattananon S, Patcharanarumol W, Thammatacharee J, Jongudomsuk P, Sirilak S (2015). Achieving universal health coverage goals in Thailand: the vital role of strategic purchasing. Health Policy Plan.

[7] Mathauer I, Dale E, Meessen B. Strategic Purchasing for Universal Health Coverage: Key Policy Issues and Questions: A Summary from Expert and Practitioners’ Discussions. World Health Organization; 2017.

[8] World Health Organization (WHO). The World Health Report 2010. WHO; 2010.

[9] Ndabarora E, Chipps JA, Uys L (2014). Systematic review of health data quality management and best practices at community and district levels in LMIC. Inf Dev.

[10] OpenRBF. https://openrbf.org. Accessed October 18, 2021.

[11] openIMIS Initiative. About openIMIS. https://openimis.org/about-openimis. Accessed October 18, 2021.

[12] Swiss TPH. What is OpenIMIS? https://www.swisstph.ch/en/about/scih/sysu/health-economics-and-financing/imis/. Accessed January 6, 2021.

[13] Federal Ministry for Economic Cooperation and Development. Open Source Software for Social Health Insurance. 2017. http://health.bmz.de/events/In_focus/Open_source_software_for_social_health_insurance/index.html. Accessed October 18, 2021.

[14] Federal Ministry for Economic Cooperation and Development. openIMIS: Co-creating a Global Good. 2020. http://health.bmz.de/what_we_do/openimis/co-creating-a-global-good/index.html. Accessed October 18, 2021.

[15] Lium JT, Tjora A, Faxvaag A (2008). No paper, but the same routines: a qualitative exploration of experiences in two Norwegian hospitals deprived of the paper based medical record. BMC Med Inform DecisMak.

[16] Shiferaw AM, Zegeye DT, Assefa S, Yenit MK (2017). Routine health information system utilization and factors associated thereof among health workers at government health institutions in East Gojjam Zone, Northwest Ethiopia. BMC Med Inform DecisMak.

[17] Ludwick DA, Doucette J (2009). Adopting electronic medical records in primary care: lessons learned from health information systems implementation experience in seven countries. Int J Med Inform.

[18] Fritz F, Tilahun B, Dugas M (2015). Success criteria for electronic medical record implementations in low-resource settings: a systematic review. J Am Med Inform Assoc.

[19] Jawhari B, Ludwick D, Keenan L, Zakus D, Hayward R (2016). Benefits and challenges of EMR implementations in low resource settings: a state-of-the-art review. BMC Med Inform DecisMak.

[20] Carnahan E, Ferriss E, Beylerian E (2020). Determinants of facility-level use of electronic immunization registries in Tanzania and Zambia: an observational analysis. Glob Health Sci Pract.

[21] Dolan SB, Alao ME, Mwansa FD (2020). Perceptions of factors influencing the introduction and adoption of electronic immunization registries in Tanzania and Zambia: a mixed methods study. Implement Sci Commun.

[22] Mangone E, Riley P, Datari K. Digital Financial Services for Health: A Global Evidence Review. Rockville, MD: USAID Local Health System Sustainability Project, Abt Associates Inc; 2021.

[23] Schreiweis B, Pobiruchin M, Strotbaum V, Suleder J, Wiesner M, Bergh B (2019). Barriers and facilitators to the implementation of eHealth services: systematic literature analysis. J Med Internet Res.

[24] Proctor E, Silmere H, Raghavan R (2011). Outcomes for implementation research: conceptual distinctions, measurement challenges, and research agenda. Adm Policy Ment Health.

[25] The United Republic of Tanzania Ministry of Health and Social Welfare. Staffing Levels for Ministry of Health and Social Welfare Departments, Health Service Facilities, Health Training Institutions and Agencies 2014-2019 (Revised). Dar es Salaam; 2014.

[26] University Consultancy Bureau (UCB) of the University of Dar Es Salaam. Household Survey Report for a Follow Up Survey for the Health Promotion and System Strengthening (HPSS). University of Dar es Salaam; 2018.

[27] Ministry of Health, Community Development, Gender, Elderly, Children (MoHCDGEC) [Tanzania Mainland], Ministry of Health (MoH) [Zanzibar], National Bureau of Statistics (NBS), Office of Chief Government Statistician (OCGS), ICF. Tanzania Demographic and Health Survey and Malaria Indicator Survey 2015-2016 [Dataset] TZIR7AFL.DTA. Dar es Salaam, Tanzania: MoHCDGEC, MoH, NBS, OCGS, and ICF; 2016.

[28] Kuunibe N, Lohmann J, Schleicher M (2019). Factors associated with misreporting in performance-based financing in Burkina Faso: implications for risk-based verification. Int J Health Plann Manage.

[29] Leys C, Ley C, Klein O, Bernard P, Licata L (2013). Detecting outliers: do not use standard deviation around the mean, use absolute deviation around the median. J Exp Soc Psychol.

[30] GEODIST: Stata Module to Compute Geographical Distances [Computer Program]. Boston College Department of Economics; 2010.

[31] Manzi F, Schellenberg JA, Hutton G (2012). Human resources for health care delivery in Tanzania: a multifaceted problem. Hum Resour Health.

[32] Kwesigabo G, Mwangu MA, Kakoko DC (2012). Tanzania’s health system and workforce crisis. J Public Health Policy.

[33] Gagnon MP, Desmartis M, Labrecque M (2012). Systematic review of factors influencing the adoption of information and communication technologies by healthcare professionals. J Med Syst.

[34] Ndima SD, Sidat M, Give C, Ormel H, Kok MC, Taegtmeyer M (2015). Supervision of community health workers in Mozambique: a qualitative study of factors influencing motivation and programme implementation. Hum Resour Health.

[35] Bradley S, Kamwendo F, Masanja H (2013). District health managers’ perceptions of supervision in Malawi and Tanzania. Hum Resour Health.

[36] Heerdegen ACS, Aikins M, Amon S, Agyemang SA, Wyss K (2020). Managerial capacity among district health managers and its association with district performance: a comparative descriptive study of six districts in the Eastern Region of Ghana. PLoS One.

[37] Fetene N, Canavan ME, Megentta A (2019). District-level health management and health system performance. PLoS One.

[38] Blacklock C, Gonçalves Bradley DC, Mickan S (2016). Impact of contextual factors on the effect of interventions to improve health worker performance in sub-Saharan Africa: review of randomised clinical trials. PLoS One.

[39] van de Klundert J, van Dongen-van den Broek J, Yesuf EM, Vreugdenhil J, Yimer SM (2018). ‘We are planning to leave, all of us’-a realist study of mechanisms explaining healthcare employee turnover in rural Ethiopia. Hum Resour Health.

[40] Naburi H, Mujinja P, Kilewo C (2017). Job satisfaction and turnover intentions among health care staff providing services for prevention of mother-to-child transmission of HIV in Dar es Salaam, Tanzania. Hum Resour Health.

[41] Leonard E, de Kock I, Bam W (2020). Barriers and facilitators to implementing evidence-based health innovations in low- and middle-income countries: a systematic literature review. Eval Program Plann.

[42] USAID. Tanzania. Results-Based Financing - Fact Sheet. 2019. https://www.usaid.gov/sites/default/files/documents/1860/03.26.2019_Results-Based_Financing.pdf. Accessed January 6, 2021.

[43] Paul E, Sossouhounto N, Eclou DS (2014). Local stakeholders’ perceptions about the introduction of performance-based financing in Benin: a case study in two health districts. Int J Health Policy Manag.

[44] Lohmann J, Wilhelm D, Kambala C, Brenner S, Muula AS, De Allegri M (2018). ‘The money can be a motivator, to me a little, but mostly PBF just helps me to do better in my job.’ An exploration of the motivational mechanisms of performance-based financing for health workers in Malawi. Health Policy Plan.

[45] Lohmann J, Muula AS, Houlfort N, De Allegri M (2018). How does performance-based financing affect health workers’ intrinsic motivation? A self-determination theory-based mixed-methods study in Malawi. Soc Sci Med.

[46] Clarke KA (2005). The phantom menace: omitted variable bias in econometric research. ConflManag Peace Sci.

[47] Huang F, Blaschke S, Lucas H (2017). Beyond pilotitis: taking digital health interventions to the national level in China and Uganda. Global Health.

[48] Kiberu VM, Matovu JK, Makumbi F, Kyozira C, Mukooyo E, Wanyenze RK (2014). Strengthening district-based health reporting through the district health management information software system: the Ugandan experience. BMC Med Inform DecisMak.

[49] Phalkey RK, Yamamoto S, Awate P, Marx M (2015). Challenges with the implementation of an Integrated Disease Surveillance and Response (IDSR) system: systematic review of the lessons learned. Health Policy Plan.

[50] Labrique A, Vasudevan L, Weiss W, Wilson K (2018). Establishing standards to evaluate the impact of integrating digital health into health systems. Glob Health Sci Pract.

[51] Labrique AB, Wadhwani C, Williams KA (2018). Best practices in scaling digital health in low and middle income countries. Global Health.

[52] Schuetze L, Srivastava S, Missenye AM, Rwezaula EJ, Stoermer M, De Allegri M (2023). Factors affecting the successful implementation of a digital intervention for health financing in a low-resource setting at scale: semistructured interview study with health care workers and management staff. J Med Internet Res.

