# Supplementary material for: What Factors Explain Low Adoption of Digital Technologies for Health Financing in an Insurance Setting? Novel Evidence From a Quantitative Panel Study on IMIS in Tanzania
Source: Int J Health Policy Manag. 2023 Feb 13;12:6896. doi: 10.34172/ijhpm.2023.6896 (PMC10125074; doi:10.34172/ijhpm.2023.6896)
Supplement: Supplementary file 1 — Calculation of Values to Estimate Outcome. [file ijhpm-12-6896-s001.pdf]

Article title: What Factors Explain Low Adoption of Digital Technologies for Health Financing in an Insurance Setting? Novel Evidence From a Quantitative Panel Study on IMIS in Tanzania

Journal name: International Journal of Health Policy and Management (IJHPM)

Authors' information: Leon Schuetze[1]*, Siddharth Srivastava[2,3], Naasegnibe Kuunibe[1,4], Elizeus Josephat Rwezaula[5], Abdallah Missenye[6], Manfred Stoermer[2,3], Manuela De Allegri[1]

[1]Heidelberg Institute of Global Health, Medical Faculty and University Hospital, University of Heidelberg, Heidelberg, Germany.

[2]Swiss Tropical and Public Health Institute (Swiss TPH), Basel, Switzerland.

[3]University of Basel, Basel, Switzerland.

[4]Faculty of Integrated Development Studies, University for Development Studies, Wa, Ghana.

[5]Health Promotion and System Strengthening Project (HPSS), Dodoma, Tanzania. 6Kongwa District Council, Dodoma, Tanzania.

(*Corresponding author: leon.schuetze@uni-heidelberg.de)

Supplementary file 1. Calculation of Values to Estimate Outcome

S1.1. calculating $\text{insured}_{f,m}$ = percentage of people insured in the facility catchment area.

Health Promotion and System Strengthening project (HPSS) staff provided data sheets with village population for every village in the 3 regions, retrieved from the district medical officers. The number of insured in each village and month was retrieved from the analytical and reporting component of IMIS (AR-IMIS). HPSS staff also provided data sheets containing the catchment villages of all facilities in the 3 regions. Data from these sources were matched to calculate a) the number of people and b) the number of insured in each facility catchment area. Percentage of people insured was then calculated.

This results in the formula

$$\text{(S1)} \quad \text{insured}_{f,m} = \frac{\text{number\_insured}_{v_1,m} + \text{number\_insured}_{v_2,m} + \ldots + \text{number\_insured}_{v_n,m}}{\text{population}_{v_1} + \text{population}_{v_2} + \ldots + \text{population}_{v_n}}$$

where $\text{insured}_{f,m}$ is the percentage of people insured via the iCHF in the catchment area of facility *f* in month *m*, $\text{number\_insured}_{v_n,m}$ is the number of people insured in village v_n in month *m* of 2017, and population_{v_n} is the population in village v_n in 2017.

Example: What is the percentage of people insured in the catchment area of facility A in January 2017?

- facility A catchment area:
    - village 1 and village 2
- number of insured in January 2017:
    - village 1: 101
    - village 2: 462
- village populations in 2017:
    - village 1: 1939
    - village 2: 3940

percentage of people insured in the catchment area of facility A in January 2017:

$$\text{insured}_{fm} = \frac{101 + 432}{1939 + 3940} = 0.096 = 9.6\%$$

S1.2. calculating utilization_{all}, utilization_{CHF} = utilization rate of public primary health care facilities for the general population (utilization_{all}) and CHF insured (utilization_{CHF}).

Utilization was defined as number of health facility visits per 100 individuals in a 4 week recall period (approximately 1 month). Demographic and Household Survey Tanzania 2014/2015[1] data was used to extract health service utilization rates for the whole population and CHF insured people in each region (Dodoma, Morogoro, Shinyanga). Extracting utilization rates on the district or even facility catchment area level was not possible due to low respondent numbers in individual districts in the DHS data. It seems reasonable to assume that CHF insured clients tend to utilize public health services over private, since their insurance only covers fees

in the public sector. This problem was taken consideration of by identifying the percentage of utilization directed to the different health services (private facility, public hospital, public dispensary/health centre, traditional healer etc) for all respondents vs CHF insured respondents. An HPSS survey of 2018[2] provided this data.

This results in the formulas

(S2)
$$\text{utilization}_{\text{all}} = \frac{\text{utilization}_{\text{all (public first level)}}}{\text{utilization}_{\text{all (all services)}}}$$

and

(S3)
$$\text{utilization}_{\text{CHF}} = \frac{\text{utilization}_{\text{CHF (public first level)}}}{\text{utilization}_{\text{CHF (all services)}}}$$

Table S1. Utilization rates by region, number of visits per 100 individuals in a 4-week period.

**region**	**$\text{utilization}_{\text{all}}$ (all services)**	**$\text{utilization}_{\text{all}}$ (public first level)**	**$\text{utilization}_{\text{CHF}}$ (all services)**	**$\text{utilization}_{\text{CHF}}$ (public first level)**
Dodoma	12.8	7.5	12.7	8.3
Morogoro	20.2	11.8	19.7	12.9
Shinyanga	16.4	9.6	25.2	16.5

References

27. Ministry of Health, Community Development, Gender, Elderly, Children (MoHCDGEC) [Tanzania Mainland], Ministry of Health (MoH) [Zanzibar], National Bureau of Statistics (NBS), Office of Chief Government Statistician (OCGS), ICF. Tanzania Demographic and Health Survey and Malaria Indicator Survey 2015-2016 [Dataset] TZIR7AFL.DTA. Dar es Salaam, Tanzania: MoHCDGEC, MoH, NBS, OCGS, and ICF; 2016.

26. University Consultancy Bureau (UCB) of the University of Dar Es Salaam. *Household Survey Report For A Follow Up Survey For The Health Promotion And System Strengthening (HPSS)*: University of Dar Es Salaam;2018.
