# Supplementary material for: What Factors Explain Low Adoption of Digital Technologies for Health Financing in an Insurance Setting? Novel Evidence From a Quantitative Panel Study on IMIS in Tanzania
Source: Int J Health Policy Manag. 2023 Feb 13;12:6896. doi: 10.34172/ijhpm.2023.6896 (PMC10125074; doi:10.34172/ijhpm.2023.6896)
Supplement: Supplementary file 2 — Descriptive Statistics. [file ijhpm-12-6896-s002.pdf]

Article title: What Factors Explain Low Adoption of Digital Technologies for Health Financing in an Insurance Setting? Novel Evidence From a Quantitative Panel Study on IMIS in Tanzania

Journal name: International Journal of Health Policy and Management (IJHPM)

Authors' information: Leon Schuetze[1]*, Siddharth Srivastava[2,3], Naasegnibe Kuunibe[1,4], Elizeus Josephat Rwezaula[5], Abdallah Missenye[6], Manfred Stoermer[2,3], Manuela De Allegri[1]

[1]Heidelberg Institute of Global Health, Medical Faculty and University Hospital, University of Heidelberg, Heidelberg, Germany.

[2]Swiss Tropical and Public Health Institute (Swiss TPH), Basel, Switzerland.

[3]University of Basel, Basel, Switzerland.

[4]Faculty of Integrated Development Studies, University for Development Studies, Wa, Ghana.

[5]Health Promotion and System Strengthening Project (HPSS), Dodoma, Tanzania. 6Kongwa District Council, Dodoma, Tanzania.

(*Corresponding author: leon.schuetze@uni-heidelberg.de)

Supplementary file 2. Descriptive Statistics

Table S2. Descriptive statistics of explanatory variables (before imputation).

variable	observations (n)	mean	SD	median	iqr	min	max
no of staff	4308	4.4	3.0	4	2	1	25
service volume	4100	448.9	427.9	329.5	341	3	7194
distance to HQ [km]	4380	39.7	30	33.2	35.6	0.5	156.5
share insured [%]	4236	15.4	13.4	12.4	13.7	0	98.8

Table S3. Claims by district, medians and inter-quartile range.

region	verified (iqr)		expected (iqr)		difference (iqr)		% difference (iqr)	
Bahi	5	(0 - 32)	39.3	(12.45 - 39.3)	14.9	(0.9 - 14.9)	77.3	(20 - 77.3)
Chamwino	1.5	(0 - 32)	38.5	(14.7 - 38.5)	19.6	(3.3 - 19.6)	94.4	(29.8 - 94.4)
Chemba	1	(0 - 24)	38	(19.5 – 38)	27.3	(10.3 - 27.3)	99.1	(58.9 - 99.1)
Dodoma CC	14.5	(0 - 43)	62.1	(30.7 - 62.1)	31.6	(3.8 - 31.6)	79.5	(34.7 - 79.5)
Kondoa DC	5	(0 - 32)	25.6	(12.5 - 25.6)	14.3	(0 - 14.3)	71.5	(2.4 - 71.5)
Kondoa TC	0	(0 - 4.5)	45.5	(20.8 - 45.5)	35.0	(18.1 - 35)	100.0	(82.5 - 100)
Kongwa	14	(0 - 38)	32.6	(16.1 - 32.6)	16.8	(-0.2 - 16.8)	64.5	(5.1 - 64.5)
Mpwapwa	0	(0 - 19.5)	13.6	(5.6 - 13.6)	4.0	(-1.9 - 4)	100.0	(-15.8 - 100)
Dodoma region	*5*	*(0 - 31)*	*34*	*(13.9 - 73)*	*18.3*	*(1.6 - 48.5)*	*83.4*	*(26.6 - 100)*
Kilombero	7	(0 - 26)	60	(23.8 – 60)	48.6	(11.7 - 48.6)	88.0	(61 - 88)
Kilosa	11	(0 - 26.5)	18.5	(5.5 - 18.5)	8.7	(-5 - 8.7)	71.5	(-6.3 - 71.5)
Malinyi	50.5	(17 - 82.5)	78.3	(39.4 - 78.3)	29.8	(1 - 29.8)	55.9	(7.1 - 55.9)
Morogoro MC	10	(0 - 27.5)	21.9	(7.5 - 21.9)	5.9	(-2.6 - 5.9)	62.8	(-17.6 - 62.8)
Mvomero	19.5	(0.5 - 50.5)	243.3	(134.1 - 243.3)	191.1	(120.6 - 191.1)	91.0	(79.3 - 91)
Ulanga	87	(47.5 - 154)	320	(145.7 – 320)	192.4	(50.3 - 192.4)	72.6	(45.8 - 72.6)
Morogoro region	*16*	*(0 - 46)*	*49.4*	*(13.3 – 152.6)*	*25.7*	*(1.3 – 119.4)*	*75.3*	*(18 - 100)*
Kahama TC	34	(17.5 - 53)	186.3	(124.6 - 186.3)	143.9	(97.7 - 143.9)	81.3	(71.4 - 81.3)
Kishapu	19	(0 - 41)	41.8	(24 - 41.8)	17.9	(5.6 - 17.9)	56.8	(16 - 56.8)
Msalala	42	(13.5 - 69)	218	(123.6 – 218)	169.5	(77.9 - 169.5)	81.9	(59.1 - 81.9)
Shinyanga DC	14	(0 - 39)	87.4	(53.5 - 87.4)	70.4	(35.8 - 70.4)	81.2	(60.5 - 81.2)
Shinyanga MC	44.5	(25 - 81.5)	234.5	(137.7 - 234.5)	172.3	(103.1 - 172.3)	77.5	(65.3 - 77.5)
Shinyanga region	*27*	*(3 - 51)*	*89.7*	*(44.2 - 189.4)*	*59.2*	*(20.1 - 144.8)*	*75.9*	*(48.1 - 96.5)*
**Total**	**13**	**(0-41)**	**49.1**	**(19.1 - 49.1)**	**27.3**	**(4.6 - 27.3)**	**77.8**	**(32.7 - 77.8)**
