# Supplementary material for: What Factors Explain Low Adoption of Digital Technologies for Health Financing in an Insurance Setting? Novel Evidence From a Quantitative Panel Study on IMIS in Tanzania
Source: Int J Health Policy Manag. 2023 Feb 13;12:6896. doi: 10.34172/ijhpm.2023.6896 (PMC10125074; doi:10.34172/ijhpm.2023.6896)
Supplement: Supplementary file 3 — Sensitivity Analysis. [file ijhpm-12-6896-s003.pdf]

Article title: What Factors Explain Low Adoption of Digital Technologies for Health Financing in an Insurance Setting? Novel Evidence From a Quantitative Panel Study on IMIS in Tanzania

Journal name: International Journal of Health Policy and Management (IJHPM)

Authors' information: Leon Schuetze[1]*, Siddharth Srivastava[2,3], Naasegnibe Kuunibe[1,4], Elizeus Josephat Rwezaula[5], Abdallah Missenye[6], Manfred Stoermer[2,3], Manuela De Allegri[1]

[1]Heidelberg Institute of Global Health, Medical Faculty and University Hospital, University of Heidelberg, Heidelberg, Germany.

[2]Swiss Tropical and Public Health Institute (Swiss TPH), Basel, Switzerland.

[3]University of Basel, Basel, Switzerland.

[4]Faculty of Integrated Development Studies, University for Development Studies, Wa, Ghana.

[5]Health Promotion and System Strengthening Project (HPSS), Dodoma, Tanzania. 6Kongwa District Council, Dodoma, Tanzania.

(*Corresponding author: leon.schuetze@uni-heidelberg.de)

Supplementary file 3. Sensitivity Analysis

S3.1. Sensitivity analysis A, with same utilization rate for all regions.

For this sensitivity analysis, the same method of calculating utilization rates was used (see formulas S2 and S3), but utilization rates were not calculated for each region but rather for all 3 regions combined. Calculated utilization rates, based on DHS data, differed significantly between regions (see Table S1). While we trust the validity of the DHS data and our calculations, we offer this sensitivity analysis to show that applying a pooled utilization rate for all regions produces similar results.

Table S5. Descriptive of outcome variables (after imputation) sensitivity analysis A.

	**percentage misreporting**			**observations**
**Outcome**	under	over	no	N
**(1) 10% difference**	80	15	4	4279
**(2) 25% difference**	76	13	11	4279
**(3) 50% difference**	66	9	25	4279
**(4) 2.5*MAD**	26	1	72	4380

Table S6. Logistic regression results sensitivity analysis A.

	**(1) 10% threshold**	**(2) 25% threshold**	**(3) 50% threshold**	**(4) 2.5*MAD**
explanatory variables				
**no. of staff**	0.051	0.051	0.057	0.105
95% CI	*(-0.04 – 0.142)*	*(-0.036 – 0.138)*	*(-0.019 – 0.133)*	*(-0.009 – 0.218)*
	P = .273	*P = .247*	*P = .144*	*P = .071*
**service volume**	0.002	0.002	0.002	0.008
95% CI	*(0.002 – 0.003)*	*(0.001 – 0.002)*	*(0.001 – 0.002)*	*(0.007 – 0.009)*
	P < .001	*P < .001*	*P < .001*	*P < .001*
**distance to HQ**	0.016	0.015	0.016	0.005
95% CI	*(0.008 – 0.024)*	*(0.008 – 0.024)*	*(0.009 – 0.023)*	*(-0.07 – 0.017)*
	P < .001	*P < .001*	*P < .001*	*P = .431*
**share insured**	0.084	0.066	0.037	0.252
95% CI	*(0.065 – 0.103)*	*(0.05 – 0.83)*	*(0.024 – 0.05)*	*(0.224 – 0.281)*
	P < .001	*P < .001*	*P < .001*	*P < .001*
**Wald Chi²**	130	115.7	97	402.5
	P < .001	*P < .001*	*P < .001*	*P < .001*
random intercepts				
**district**	0.071	0.103	0	0.314
95% CI	*(0.006 –0.9)*	*(0.013 – 1.84)*	*(0 – 0)*	*(0.052 – 1.907)*
**facility**	2.838	2.851	2.928	4.594
95% CI	*(2.172 – 3.708)*	*(2.213 – 3.672)*	*(2.335 – 3.671)*	*(3.307 – 6.282)*
**N**	4279	4279	4279	4380

S3.2. Sensitivity analysis B, with a different method of computing expected claims.

For this analysis, $\text{expected}_{\text{fm}}$ was estimated using the formula

(S4) $$\text{expected}_{\text{fm}} = \text{number\_insured}_{\text{fm}} \times \text{utilization}_{\text{CHF}}$$

where $\text{expected}_{\text{fm}}$ is the number of expected claims for facility *f* in month *m*, $\text{number\_insured}_{\text{fm}}$ is the number of people insured in the facility catchment area of facility *f* in month *m*, and $\text{utilization}_{\text{CHF}}$ is the utilization rate of CHF insured people in the region. Conceptually, this formula relies on the number of insured people in the facility catchment area as grounds for estimation, while formula (1) additionally considers the actual number of visits to the facility in a given time period. Therefore, we considered formula (1) to be more robust and precise. Nevertheless, we offer this analysis as an alternative.

Table S7. Descriptive of outcome variables (after imputation) sensitivity analysis B.

	**percentage misreporting**			**observations**
**outcome**	under	over	no	N
**(1) 10% difference**	88	10	3	4279
**(2) 25% difference**	85	8	7	4279
**(3) 50% difference**	76	6	18	4279
**(4) 2.5*MAD**	27	0	72	4380

Table S8. Logistic regression results sensitivity analysis B.

	(1) 10% threshold	(2) 25% threshold	(3) 50% threshold	(4) 2.5*MAD
explanatory variables				
**no. of staff**	0.404	0.322	0.244	*not converged*
95% CI	*(0.208 – 0.6)*	*(0.154 – 0.489)*	*(0.03 – 0.358)*	
	P < .001	*P < .001*	*P < .000*	
**service volume**	-0.001	-0.001	-0.001	*not converged*
95% CI	*(-0.002 – -0.001)*	*(-0.003 – 0.021)*	*(-0.001 – 0)*	
	P < .001	*P < .001*	*P < .001*	
**distance to HQ**	0.008	0.009	0.008	*not converged*
95% CI	*(-0.005 – 0.02)*	*(-0.003 – 0.021)*	*(-0.002 – 0.018)*	
	P = .224	*P = .147*	*P = .097*	
**share insured**	0.041	0.038	0.025	*not converged*
95% CI	*(0.02 – 0.062)*	*(0.019 – 0.056)*	*(0.01 – 0.039)*	
	P < .001	*P < .001*	*P = .001*	
**Wald Chi²**	46.3	39.5	40.1	*not converged*
	P < .001	*P < .001*	*P < .001*	
random intercepts				
**district**	2.221	1.669	0.825	*not converged*
95% CI	*(0.824 –5.985)*	*(0.639 – 4.36)*	*(0.322 – 2.113)*	
**facility**	4.402	4.345	3.588	*not converged*
95% CI	*(3.187 – 6.079)*	*(3.205 – 5.891)*	*(2.768 – 4.651)*	
**N**	4279	4279	4279	*not converged*

S3.3. Sensitivity analysis C, without imputation of missing values.

For our main analysis, we used a single imputation approach to impute missing values. To verify the validity of this approach, we performed this sensitivity analysis, where missing values were dropped, and no imputation was performed. The results are consistent with the main analysis, supporting the notion that values were missing at random and imputation was justified.

Table S9. Descriptive of outcome variables sensitivity analysis C.

	**percentage misreporting**			**observations**
**outcome**	under	over	no	N
**(1) 10% difference**	82	15	4	3800
**(2) 25% difference**	77	12	10	3800
**(3) 50% difference**	67	9	24	3800
**(4) 2.5*MAD**	28	1	71	3901

Table S10. Logistic regression results sensitivity analysis C.

	(1) 10% threshold	(2) 25% threshold	(3) 50% threshold	(4) 2.5*MAD
explanatory variables				
**no. of staff**	0.045	0.065	0.080	0.117
95% CI	*(-0.059 – 0.149)*	*(-0.037 – 0.167)*	*(-0.013 – 0.174)*	*(-0.023 – 0.257)*
	P = .392	*P = .213*	*P = .093*	*P = .102*
**service volume**	0.002	0.002	0.002	0.008
95% CI	*(0.001 – 0.003)*	*(0.001 – 0.003)*	*(0.001 – 0.002)*	*(0.007 – 0.009)*
	P < .001	*P < .001*	*P < .001*	*P < .001*
**distance to HQ**	0.014	0.012	0.015	0.002
95% CI	*(0.005 – 0.023)*	*(0.004 – 0.021)*	*(0.007 – 0.024)*	*(-0.012 – 0.015)*
	P < .001	*P = .006*	*P < .001*	*P = .813*
**share insured**	0.089	0.077	0.054	0.266
95% CI	*(0.068 – 0.11)*	*(0.059 – 0.095)*	*(0.039 – 0.069)*	*(0.233 – 0.299)*
	P < .001	*P < .001*	*P < .001*	*P < .001*
**Wald Chi²**	112.2	116.9	103.2	318.3
	P < .001	*P < .001*	*P < .001*	*P < .001*
random intercepts				
**district**	0.411	0.465	0.242	2.885
95% CI	*(0.099 –1.702)*	*(0.112 – 1.925)*	*(0.048 – 1.227)*	*(1.232 – 6.755)*
**facility**	2.442	2.546	2.798	3.725
95% CI	*(1.808 – 3.298)*	*(1.922 – 3.371)*	*(2.173 – 3.603)*	*(2.564 – 5.413)*
**N**	3733	3733	3733	3792
